# Quantification of tumour-infiltrating immune cells through deconvolution of DNA methylation data in Ewing sarcomas

**DOI:** 10.3389/freae.2024.1427399

**Published:** 2024-09-04

**Authors:** Sara Patrizi, Silvia Vallese, Lucia Pedace, Claudia Nardini, Alessandra Stracuzzi, Sabina Barresi, Isabella Giovannoni, Luana Abballe, Celeste Antonacci, Ida Russo, Angela Di Giannatale, Rita Alaggio, Franco Locatelli, Giuseppe Maria Milano, Evelina Miele

**Affiliations:** ^1^ Onco-Hematology, Cell Therapy, Gene Therapies and Hemopoietic Transplant, Bambino Gesù Children’s Hospital, IRCCS, Rome, Italy; ^2^ Pathology Unit, Bambino Gesù Children’s Hospital, IRCCS, Rome, Italy

**Keywords:** Ewing sarcoma, DNA methylation, immune microenvironment, deconvolution, immunotherapy

## Abstract

Ewing Sarcomas (EWS, OMIM^#^612219) presents a major challenge in pediatric oncology due to its aggressive nature and poor prognosis, particularly in metastatic cases. Genetic fusions involving the *EWSR1* gene and ETS family transcription factors are common in EWS, though other rarer fusions have also been identified. Current standard techniques like immunohistochemistry have failed to fully characterize the immune tumor microenvironment of EWS, hindering insights into tumor development and treatment strategies. Recent efforts apply gene expression datasets to quantify tumor-infiltrating immune cells in EWS. Similar deconvolution techniques can be also applied to DNA methylation (DNAm) arrays, which are much more stable compared to RNA-based methods. This study aims to characterize immune cell infiltration in EWS using DNAm array data. We collected 32 EWS samples from 32 consecutive patients referred to Bambino Gesù Children’s Hospital. DNAm analysis was performed by EPIC arrays; data loading, normalization, deconvolution and survival analysis were then performed in R programming environment. We observed a higher content of dendritic cells and longer overall survival in samples with *EWSR1::FLI1* translocation compared to samples with rarer fusions. Moreover, T-memory lymphocytes and monocytes emerged as a significant predictor of overall survival. This study underscores the potential of DNAm arrays in providing robust insights into EWS immune profiles, offering a promising avenue for future research. Further investigations with larger cohorts are warranted to validate these findings and explore additional immune cell types influencing EWS outcomes.

## 1 Introduction

Ewing sarcomas (EWS) most often affect the bones and soft tissues in children and young adults and are known for their aggressive nature and their dismal prognosis when diagnosed in the metastatic stage. From a histological perspective, these tumors fall within the category of “undifferentiated small round blue cell sarcomas of bone and soft tissue (WHO 2020 and WHO 2022).” They consist of small, monotonous round cells exhibiting nuclei with finely dispersed chromatin and thin rim of cytoplasm. The cells are arranged in sheets with occasional rosette formation and frequent necrotic areas (Yoshida, 2023; Sbaraglia et al., 2021; Pfister et al., 2022). The differential diagnosis from other Ewing-like sarcomas (ELS), requires molecular characterization (Llombart-Bosch et al., 2009; Italiano et al., 2012; Antonescu, 2014). EWS are characterized by genetic fusions involving an *EWS* gene and an ETS family transcription factor (Delattre et al., 1992). The most frequent fusion involves *EWSR1* (OMIM*133450) and *FLI1* (OMIM*193067), but other rarer rearrangements with the same biological behavior have also been identified (Sorensen and Triche, 1996; Arvand and Denny, 2001).

So far, standard techniques like immunohistochemistry have been unsuccessful in providing a complete characterization of the immune tumour microenvironment of EWS (Paydas et al., 2016; Berghuis et al., 2011). Some efforts have been made using single-cell RNA sequencing (Visser et al., 2023; Cillo et al., 2022), a technique that still presents several challenges and limitations (Robinson et al., 2022). An accurate quantification of tumour-infiltrating immune cells (TIICs), however, would be valuable to shed light on the development and aggressiveness of these tumours and provide new avenues for treatment (Evdokimova et al., 2022).

Recently, Stahl et al. analysed microarray gene expression datasets of primary EWS samples using the CIBERSORT deconvolution algorithm to determine the proportion of 22 immune cell types within bulk tumour tissue (Stahl et al., 2019).

Such deconvolution can be also inferred from DNA methylation (DNAm) arrays, which are more robust than gene expression arrays given the higher stability of DNA compared with RNA (Alberts et al., 2002). Moreover, DNAm data is known to be less influenced by copy number variation than gene expression (Stranger et al., 2007; Houseman et al., 2009), a relevant concern in the field of oncology where most samples display copy number variations. Lastly, DNAm arrays are currently widely used to aid soft tissue sarcoma diagnosis through machine-learning based classifiers (Koelsche et al., 2021), while RNA sequencing currently lacks diagnostic applications. Thus, performing both diagnostic profiling and tumor microenvironment characterization on the same data appears convenient.

The aim of this study is to characterize the relative abundance of immune cells infiltrated in EWS using DNAm array, a technique that is already implemented in the diagnostic workflow of challenging sarcoma cases at our Institution, and to correlate it with patients’ survival.

## 2 Methods

We selected 32 EWS samples from 32 patients with challenging diagnosis referred to our institution and analysed for DNAm profiling (from 2020 to 2024) and, after acquiring informed consent from either the patient or their legal guardian, retrieved their follow-up information from clinical records (study protocol number 2126_OPBG_2020, reviewed and approved by the Bambino Gesù Children’s Hospital Ethical Committee). We reviewed 30/32 cases with available tissue, evaluating parameters that could affect the tumor purity. In detail, we quantified the percentage of inflammatory cells, by CD45 antibody staining (mouse monoclonal antibody, clone 2B11+PD7/26, ready to use, Dako) performed on DAKO OMNIS platform scoring it 0 (<1%), 1 (1–2%), 2 (3–5%), or 3 (>5%); the presence (Yoshida, 2023) or absence (0) of necrosis and collagenized stroma. We calculated a final “total infiltration score” from the sum of the previously described parameters and correlated it with the tumor purity calculated from DNAm deconvolution data.

We extracted both RNA (by ReliaPrep™ FFPE Total RNA kit, Promega) and DNA (by MagPurix FFPE DNA Extraction Kit, Zzinexts, Life Science Corporation, New Taipei City, Taiwan) from unstained sections of paraffin blocks containing at least 70% tumour cells.

We used either whole-transcriptome RNA sequencing (in 3/32 cases), RT-PCR or Archer FusionPlex custom panel to detect the presence of gene fusions, as described previously (Patrizi et al., 2024).

We carried out DNA methylation (DNAm) analysis using the Human MethylationEPIC v1.0 and v2.0 BeadChip arrays (Illumina), according to the manufacturer’s instructions, as previously reported (Salgado et al., 2023).

We analyzed raw BeadChip data with R package ChAMP (version 2.26.0) (Tian et al., 2017), that was chosen because it conveniently integrates existing analysis tools into a single pipeline. We loaded v1.0 and v2.0 data separately with method “minfi” (Aryee et al., 2014) and filtering out probes with detection *p*-value > 0.01. Then, we merged v1.0 and v2.0 raw beta values according to probe name and genomic position. We normalized the merged raw methylation beta values using the BMIQ method (Teschendorff et al., 2013), confirmed the absence of confounding batch factors with function champ. SVD, and performed immune cell deconvolution through package MethylResolver (Arneson et al., 2020). This deconvolution method can resolve fractions of 11 immune cell types, both in terms of relative abundance to the total amount of immune cells and of absolute abundance scaled according to tumor purity (the percentage of tumor cells in the total tissue), without the need to develop a cancer-specific signature.

We used two-tailed heteroschedastic Student’s T-test to evaluate the statistical significance of differences in immune cell content between sample groups, and Kaplan-Meier analysis performed with R package survival (https://CRAN.R-project.org/package=survival) to assess the relationship between immune cell content and overall survival. For Kaplan-Meier analysis, we determined the optimal cutpoint for each continuous variable using function surv_cutpoint from package survminer (https://CRAN.R-project.org/package=survminer), that uses the maximally selected rank statistics from the maxstat R package to divide a continuous variable in order to obtain the most significant correlation with survival.

## 3 Results

The study cohort includes 32 tumors with histological diagnosis of Ewing sarcoma from 32 consecutive patients less than 30 years old at the time of diagnosis. Patients’ characteristics are summarized in Table 1. The cohort included 17 females (53%) and 15 males (47%). The median age was 11 years old. Most samples (28/32) had the *EWSR1::FLI1* fusion, while 4 had other translocations involving *EWSR1* (2 *EWSR1::ERG*, 2 *EWSR1::ETV4*). In 30/32 cases the tissue was obtained at the moment of diagnosis of the primary tumor. One case was a very late relapse, while for 1 this information was not available (Supplementary Table S1). The level of immune infiltration observable through histological review was generally extremely low and did not allow the quantification of specific cell types (Supplementary Table S1; Supplementary Figure S1). We also evaluated further histological aspects that could influence tumor purity, observing necrosis/haemorragy in 15/30 and fibrosis in 13/30 cases. However, we observed no correlation between the total infiltration score and DNAm-inferred tumor purity (correlation score 0.26).

**TABLE 1:** Characteristics of the selected EWS samples, including gender, age at diagnosis, status at last follow up (DOD = Dead of Disease, NA = Not Available), translocation, and translocation type.

Case #	Gender (M/F)	Age at diagnosis (years)	Status at last follow up	Overall survival (years)	Translocation
1	M	5	NA	NA	EWSR1::ETV4
2	F		NA	NA	EWSR1::FLI1
3	F	14	DOD	1.58	EWSR1::FLI1
4	M	16	ALIVE	4.17	EWSR1::FLI1
5	M	12	ALIVE	1.42	EWSR1::FLI1
6	F	5	ALIVE	4.25	EWSR1::FLI1
7	M	11	DOD	3.75	EWSR1::FLI1
8	F	12	DOD	2.08	EWSR1::FLI1
9	F	17	ALIVE	4.92	EWSR1::FLI1
10	F	9	NA	NA	EWSR1::FLI1
11	F	34	NA	NA	EWSR1::FLI1
12	M	10	DOD	0.08	EWSR1::FLI1
13	M	6	DOD	1.08	EWSR1::ERG
14	F	18	DOD	0.58	EWSR1::FLI1
15	M	11	NA	NA	EWSR1::FLI1
16	F	4	NA	NA	EWSR1::FLI1
17	F	9	NA	NA	EWSR1::FLI1
18	F	27	NA	NA	EWSR1::FLI1
19	F	10	DOD	0.58	EWSR1::ERG
20	F	13	ALIVE	1.25	EWSR1::FLI1
21	F	16	ALIVE	1.17	EWSR1::FLI1
22	F	10	ALIVE	1.08	EWSR1::FLI1
23	M	1	ALIVE	0.83	EWSR1::FLI1
24	M	2	ALIVE	0.83	EWSR1::ETV4
25	M	6	ALIVE	0.67	EWSR1::FLI1
26	M	9	ALIVE	0.92	EWSR1::FLI1
27	M	7	ALIVE	0.67	EWSR1::FLI1
28	M	11	DOD	2.58	EWSR1::FLI1
29	M	16	ALIVE	0.42	EWSR1::FLI1
30	F	13	ALIVE	0.25	EWSR1::FLI1
31	M	15	ALIVE	0.50	EWSR1::FLI1
32	F	5	ALIVE	4.83	EWSR1::FLI1

The immune cell deconvolution returned the abundance of 11 cell types, both relatively to the total amount of immune cells (relative abundance) and to the total tissue (absolute abundance).

Across the whole cohort, the most abundant cell types were T-memory lymphocytes (“Tmem,” mean percentage 37.49%), T-naïve lymphocytes (mean percentage 20.56%), neutrophils (mean percentage 14.10%), and natural killer (NK) cells (mean percentage 10.44%) (Figures 1A, B; Supplementary Table S1). When compared to *EWSR::FLI1* rearranged, samples with rarer translocations displayed a significantly lower mean content of dendritic cells, both absolute (0% versus 0.61%) and relative to the immune component (0% versus 4.37%) (Figures 2A, B; Supplementary Table S1).

**FIGURE 1:**
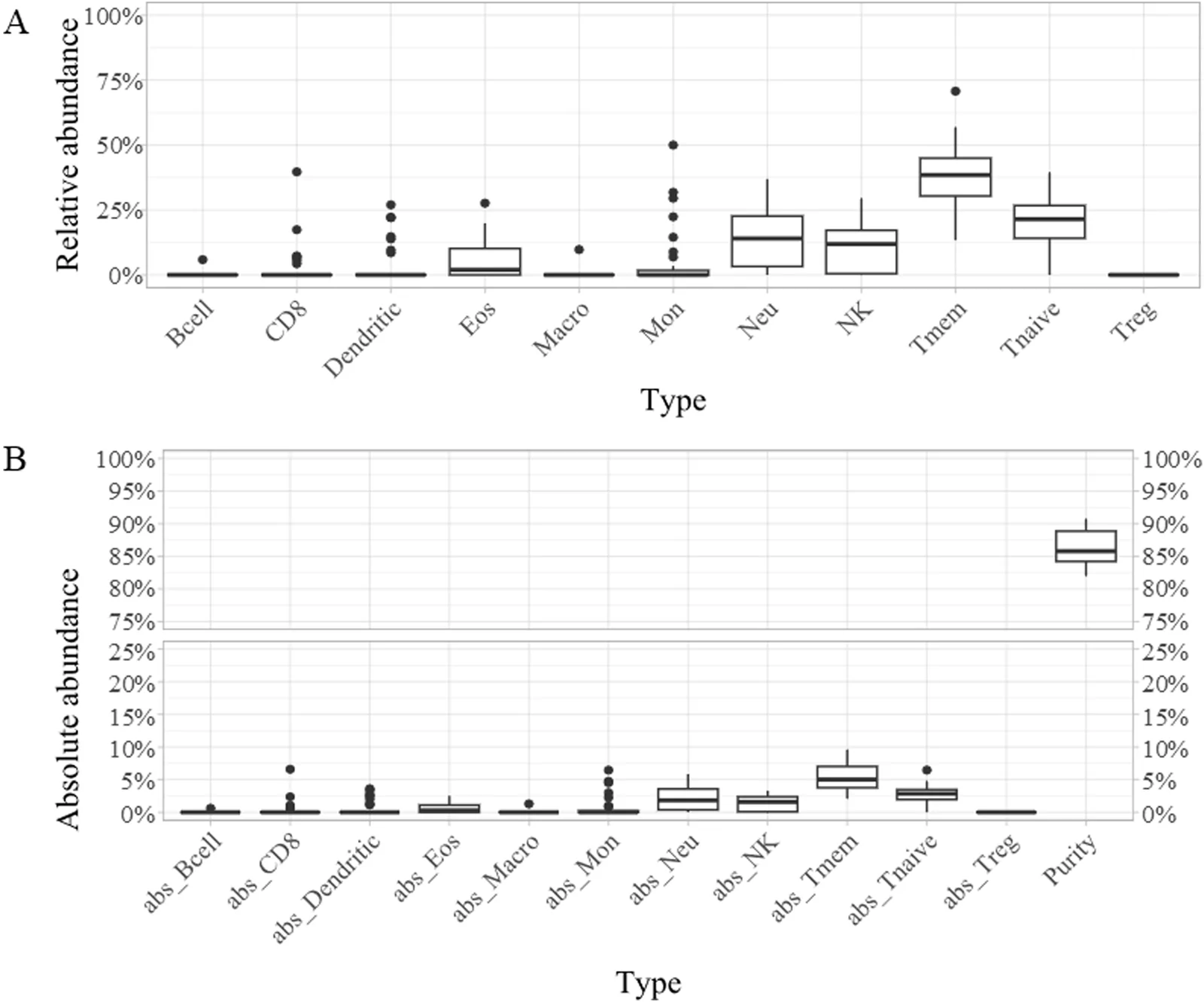
Boxplots representing the relative **(A)** and absolute [“abs,” **(B)**] abundance of each of the 11 cell types detected across the whole cohort: B cells (Bcell), CD8 lymphocytes (CD8), dendritic cells (Dendritic), eosinophiles (Eos), macrophages (Macro), monocytes (Mon), neutrophils (Neu), natural killer cells (NK), T-memory lymphcytes (Tmem), T-naive lymphocytes (Tnaive), T-regulatory lymphocytes (Treg), tumor purity (Purity).

**FIGURE 2:**
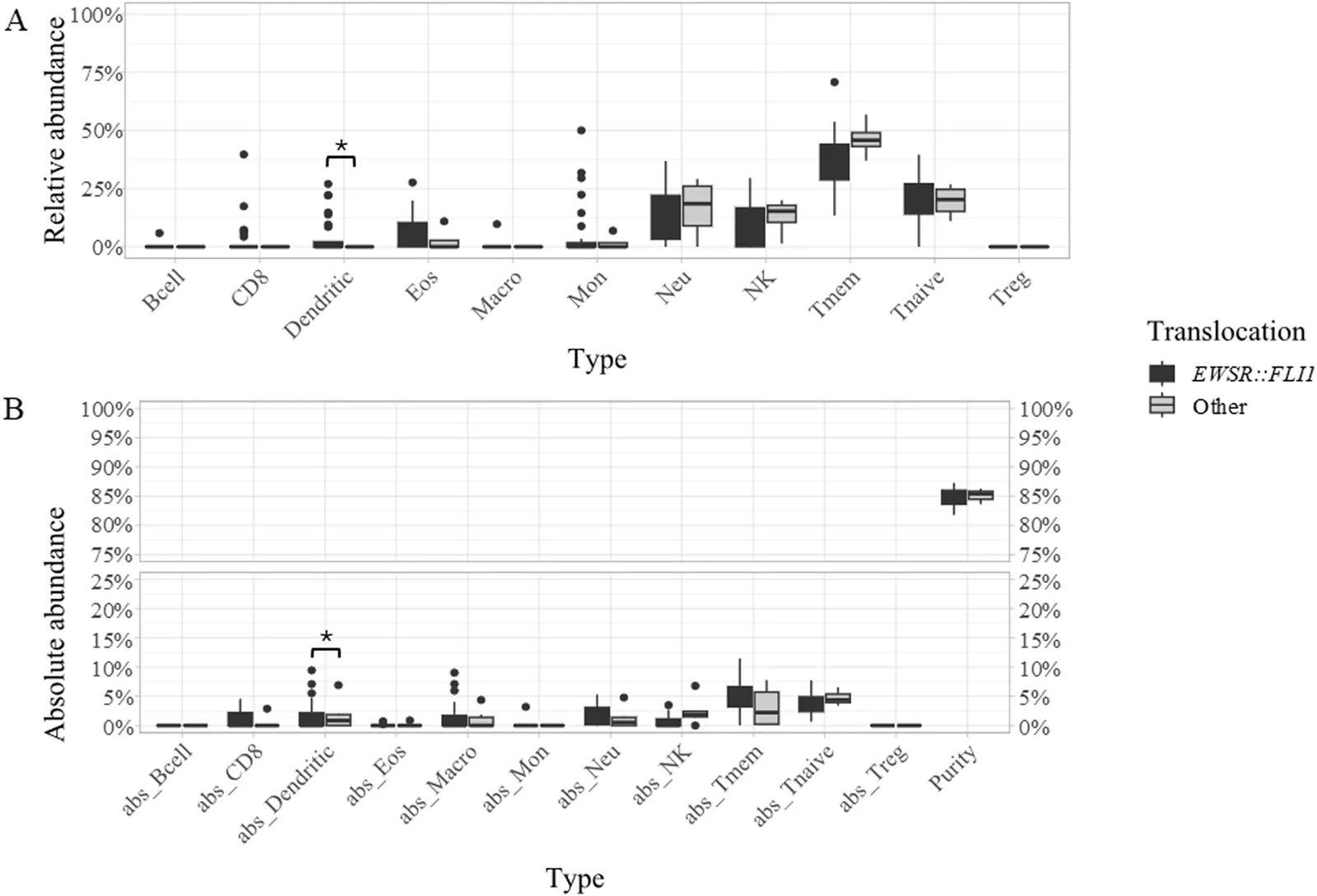
Boxplots representing the relative **(A)** and absolute [“abs,” **(B)**] abundance of each of the 11 cell types detected in samples with EWSR::FLI1 translocation (dark grey) or other rearrangements (light grey). The immune cell types include B cells (Bcell), CD8 lymphocytes (CD8), dendritic cells (Dendritic), eosinophiles (Eos), macrophages (Macro), monocytes (Mon), neutrophils (Neu), natural killer cells (NK), T-memory lymphcytes (Tmem), T-naive lymphocytes (Tnaive), T-regulatory lymphocytes (Treg), tumor purity (Purity). Statistically significant differences between patient groups are labeled by an asterisk.

Follow-up was available for 24/32 patients (Table 1). Samples with *EWSR1::FLI1* translocation showed a significantly longer overall survival than samples with rarer alterations (Supplementary Table S1). Moreover, Kaplan-Meier analysis revealed two variables significantly associated with overall survival in the whole cohort. One was the absolute content of T-memory lymphocytes, that correlated with better outcomes (Bonferroni *p*-value = 0.04) when it was higher than 2.05% (Figure 3A), and the other was the absolute content of monocytes, that was associated with higher overall survival when lower than 0.82% (Figure 3B; Supplementary Table S2). Moreover, patients with tumor purity lower than 83.73% had a higher survival probability, although not significantly (Bonferroni *p*-value = 0.11) (Supplementary Table S2).

**FIGURE 3:**
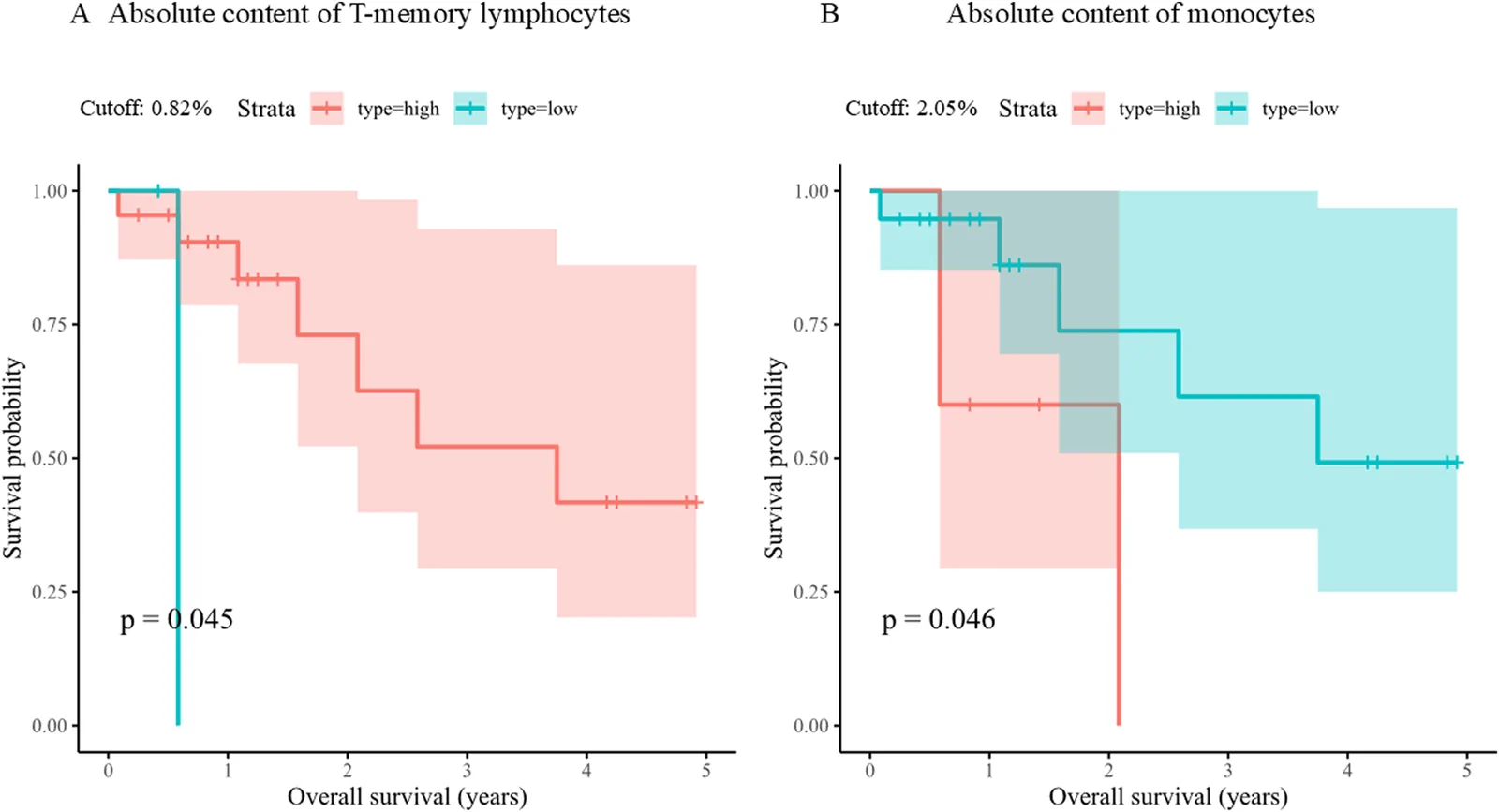
Kaplan-Meier plots of overall survival based on absolute content of T-memory lymphocytes **(A)** and monocytes **(B)**. For each continuous variable, the cohort was divided in two groups (Strata) according to its value (high or low) relatively to its optimal cutpoint (Cutoff).

## 4 Discussion

Our cohort included 32 patients with a median age of 11 years at diagnosis, which reflects the age of peak incidence of Ewing sarcoma. However, we did not observe the same male prevalence that is reported in literature (Durer et al., 2024). The immune cell deconvolution showed that the most abundant cell types across the cohort were lymphocytes, representing 60.63% of all TIICs considering Tmem, T-naïve and CD8 lymphocytes (2.54%), followed by neutrophils (mean percentage 14.10%). This result differs from the prevalence of macrophages detected from RNA sequencing data by Stahl et al. Lymphocyte prevalence has been reported as a characteristic of tumours with “hot” immune microenvironment (Wang et al., 2023), whereas Ewing sarcoma is known as a “cold” tumour, with low expression of neoantigens and low leucocyte infiltration (Evdokimova et al., 2022). The low and homogenous absolute immune cell content measured for each sample in our cohort supports the latter concept.

In our cohort, samples with *EWSR1::FLI1* translocation presented significantly longer overall survival and higher dendritic cells content (both relative and absolute) than the cases with different translocations. Since dendritic cells are known to play an important role in presenting tumour antigens to the immune system, and are often downregulated in metastatic and aggressive cancers (Del Prete et al., 2023), these two observations could be correlated. However, functional studies on a larger cohort would be needed to reach a definite conclusion.

We also found a significant correlation of longer overall survival with higher relative Tmem content, which supports previous reports of their important anti-tumor role (Liu et al., 2020). In some contexts, however, the presence of tissue-resident memory T cells has been related to a stronger recruitment of other immune cells, causing a loss of MHC class I protein expression on tumour cells and thus favoring their escape from immune response (Weeden et al., 2023). Therefore, the role of Tmem in the immune microenvironment of EWS appears intriguing and worthy of further investigations. Patients with lower tumor purity (and in consequence with higher immune infiltrate) also had better outcomes, albeit not significantly, which leads us to hypothesize that, even in a cold tumor microenvironment, an immune response can limit the cancerous cell proliferation.

Our study shows that TIICs proportions in EWS can be inferred from DNA methylation data, thus providing an alternative to traditional techniques or gene expression arrays. A weakness of our study is the lack of correlation between DNAm-inferred tumor purity and our total infiltration score derived from histopathological evaluation. We can speculate that this could be due to the selection of a different histological section for DNA methylation and histological review, or to method-specific biases. Further specific studies on larger sample groups are needed, to better assess the correlation between deconvolution results from different data sources and histopathology, and to confirm the cell types we identified as directly or inversely correlated with survival.

## Data Availability

The data presented in the study are deposited in the NCBI GEO repository, accession number GSE276012.
