# Supplementary material for: Quantification of tumour-infiltrating immune cells through deconvolution of DNA methylation data in Ewing sarcomas
Source: Front Epigenet Epigenom. 2024 Sep 4;2:1427399. doi: 10.3389/freae.2024.1427399 (PMC13639312; doi:10.3389/freae.2024.1427399)
Supplement: Supplementary file 1 [file Image1.PDF]

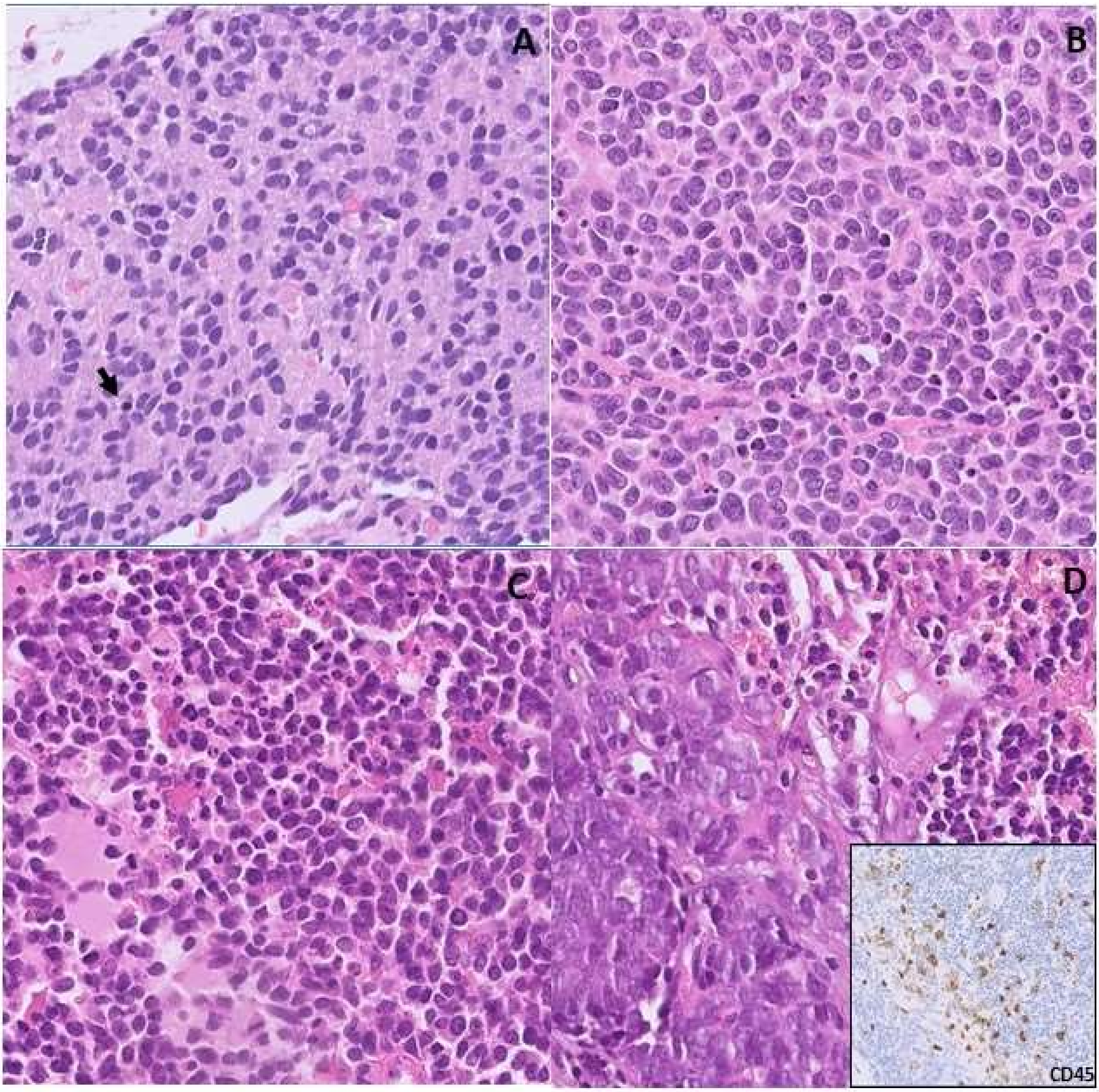

Supplementary Figure 1: Histology of TIIC classes. In the first class (A, score 1) only rare lymphocytes (arrow) intermixed with neoplastic cells can be seen. In the second class (B, score 2) a scattered infiltrate with granulocytes and lymphocytes can be appreciated. In the third and fourth class the inflammatory infiltrate is mild (C, score 3) or moderate (D, score 4) with the presence of macrophages better highlighted by immunohistochemical staining for CD45 (D inset).
